# Use of extracorporeal shockwave therapy combined with standard rehabilitation following anterior cruciate ligament reconstruction: a systematic review with meta-analysis

**DOI:** 10.1186/s12891-025-08277-9

**Published:** 2025-01-23

**Authors:** Jaehyung Shin, Hye Chang Rhim, James Kim, Raymond Guo, Ramy Elshafey, Ki-Mo Jang

**Affiliations:** 1 Foot & Ankle Research and Innovation Lab (FARIL), Department of Orthopaedic Surgery, Massachusetts General Hospital, Harvard Medical School, Boston, MA 02114 USA; 2 Department of Physical Medicine and Rehabilitation, Harvard Medical School, Spaulding Rehabilitation Hospital, Boston, MA 02114 USA; 3 Chobanian and Avedisian School of Medicine, Boston University, Boston, MA 02118 USA; 4 Department of Orthopedics & Rehabilitation, Tufts Medical Center, Boston, MA 02111 USA; 5 Department of Orthopaedic Surgery, Anam Hospital, Korea University College of Medicine, Seoul, 02841 Republic of Korea; 6 Knee Joint & Sports Medicine, Department of Orthopedic Surgery, Anam Hospital, Korea University College of Medicine, Anam-dong 5-ga Seongbuk-gu, Seoul, 02841 South Korea

**Keywords:** Extracorporeal shockwave therapy, Anterior cruciate ligament, Anterior cruciate ligament reconstruction, Postoperative rehabilitation, Knee joint

## Abstract

**Background:**

Anterior cruciate ligament (ACL) injuries are one of the most common sports injuries, accounting for approximately 50% of knee-related injuries. Extracorporeal shockwave therapy (ESWT), in the form of the radial (R-SWT) or focused shockwave (F-SWT), has been shown effective in treating various orthopaedic conditions. Recently, studies have investigated whether ESWT combined with standard rehabilitation may improve outcomes following anterior cruciate ligament reconstruction (ACLR). Therefore, this study aimed to determine whether ESWT can improve clinical outcomes following ACLR.

**Methods:**

This study followed the Preferred Reporting Items for Systematic Reviews and Meta-Analyses (PRISMA). We searched PubMed, Embase, and Web of Science and included studies involving ESWT treatment following ACLR, which consisted of randomized controlled trials (RCTs) and cohort studies. Two authors independently extracted the outcome measurements and used a revised Cochrane risk-of-bias tool (RoB 2) for RCTs and the Risk of Bias in Non-randomised Studies of Interventions (ROBINS-I) for a cohort study to assess the risk of bias. A random effects pairwise meta-analysis was used to compare patient-reported outcomes between ESWT and controlled treatments.

**Results:**

Five studies (Level I: 4; Level II: 1) with 242 participants (male: 167; female: 75) were included. Regarding the patient-reported outcomes, the risk of bias for all RCTs was ‘high’ and ‘serious’ for a non-randomized study. The meta-analysis demonstrated that the Lysholm scores were significantly higher in ESWT groups than those of controls at 12 months (Weighted mean difference [WMD]: 7.037, 95% confidence interval [CI]: 6.172–7.902, I^2^: 0%) and 24 months (WMD: 5.463, 95% CI: 2.870–8.056, I^2^: 0%). Furthermore, the International Knee Documentation Committee (IKDC) scores were also significantly higher in the ESWT group than that of the control at 12 months (WMD: 6.371, 95% CI: 3.397–9.344, I^2^: 68.8%). However, the WMDs for these outcomes between the two groups did not exceed the minimal clinically important difference (MCID).

**Conclusion:**

Based on the meta-analyses performed with a few studies, ESWT combined with standard rehabilitation may potentially lead to better patient-reported outcomes. However, these differences may not be clinically significant. Further high-quality studies are needed to confirm our review’s findings.

**Supplementary Information:**

The online version contains supplementary material available at 10.1186/s12891-025-08277-9.

## Background

Anterior cruciate ligament (ACL) injuries are known to be one of the most prevalent knee injuries across various sports activities, accounting for approximately 50% of knee-related injuries [1]. ACL injuries are commonly treated with ACL reconstruction (ACLR) to regain knee stability and allow athletes to return to sports [2]. Moreover, rehabilitation following ACLR, such as accelerated [3] and supervised [3, 4] rehabilitation, is crucial to further improve surgical outcomes and rates of return to sports [3]. Some of the rehabilitation components following ACLR include cryotherapy, open kinetic chain exercises, neuromuscular electrical stimulation, electromyography biofeedback, and whole-body vibration [3].

Extracorporeal shockwave therapy (ESWT) is a non-invasive rehabilitative modality that has been shown effective in treating various orthopaedic conditions [5]. ESWT devices emit acoustic waves to transmit energy through body tissues, thereby generating interstitial and extracellular reactions [6], such as pain relief [6, 7], enhanced collagen production, and increased cell growth and tissue repair [7]. There are currently two main types of ESWT: radial (R-SWT) and focused shockwave therapy (F-SWT). The mechanism of F-SWT involves converging the energy pulses with the high energy at a specific focal point in the body, attaining the most elevated pressure at this location [7, 8]. This delivery of energy can be achieved electrohydraulically, electromagnetically, or piezoelectrically, which are the three main methods F-SWT is performed by commercial devices [7]. In contrast, R-SWT works by dispersing those pressure fields, with the highest pressure detected at the origin [8] and decreasing as the fields transit further from the origin [7]. Given these characteristics, F-SWT is often used to target deeper structures of the body, whereas R-SWT is utilized for superficial structures [7]. Research in global trends on ESWT identified that ESWT had been increasingly used to treat musculoskeletal injuries, including conditions following orthopaedic surgeries, to optimize recovery [9]. Recently, studies have been conducted to investigate whether ESWT combined with standard rehabilitation may improve outcomes following ACLR. However, there has yet to be a review that synthesized the literature evaluating the efficacy of ESWT in patients undergoing ACLR. Therefore, this systematic review aimed to determine whether ESWT can improve clinical outcomes following ACLR.

## Methods

### Systematic review registration

This systematic review study has been done in accordance with the Preferred Reporting Items for Systematic Reviews and Meta-Analyses (PRISMA) [10]. The protocol of this review has been prospectively registered with INPLASY (2023120116).

### Search strategy and inclusion and exclusion criteria

We searched PubMed (NLM), Embase (Elsevier), and Web of Science (Clarivate) for randomized controlled trials (RCTs) and comparative studies on January 20, 2024 (Additional file 1). Reference lists of relevant articles were also reviewed. We included the studies involving ESWT treatment following ACLR, consisting of randomized controlled trials (RCTs) and cohort studies that have either control or comparators and have a full text available. We excluded the abstracts published or submitted to conferences, review articles, studies related to the genetic and molecular level research and animals, commentaries, case reports, case series, responses to letters, and letters to the editor. We further restricted studies published in English, as prior literature reported that language restriction does not lead to systemic bias [11]. The level of evidence of each study was evaluated based on The Oxford 2011 Levels of Evidence [12].

### Data extraction

Data from selected studies were independently extracted by two authors (JS and HCR) and put into a template. The template consisted of author information, country of study publication, study design, population (group, sample size, age, and sex), duration of symptom, ESWT parameters, ACLR graft choice, co-intervention, comparators, outcome measurements (patient-reported outcomes and functional scores and outcomes), duration of follow-up, main findings, return to sports or activities, and complication or adverse events. The primary outcomes were Lysholm, International Knee Documentation Committee (IKDC), and visual analogue scale (VAS) scores and magnetic resonance imaging (MRI) and radiographic outcomes.

### Risk of bias assessment

For RCTs, a revised Cochrane risk-of-bias tool (RoB 2) was utilized to assess the risk of bias, which evaluates the bias in five different domains: the process of randomization, deviations from the interventions intended, unavailable outcome data, outcome measurement process, and selection of reported findings from the study [13]. If all domains had a ‘low’ risk of bias, the overall risk of bias of the study was considered ‘low’. If there were up to three domains that were of ‘some concern’, then the study was considered ‘some concerns’. Lastly, if a study had at least one ‘high’ risk domain or three or more domains that were of ‘some concern’, then it was considered to be a ‘high’ overall risk of bias.

Regarding the non-randomized comparative studies, the Risk of Bias in Non-randomised Studies of Interventions (ROBINS-I) was implemented to evaluate the risk of bias, which determines the overall risk of bias of the study based on seven different domains: confounding factors, selection of the study participants, intervention classifications to the participants, deviations from the interventions intended, unavailable outcome data, outcome measurement process, and selection of the reported findings from the study [14]. If all domains were considered to have a low risk of bias, the overall risk of bias of the study was determined to be ‘low’. If all domains were assessed as either ‘low’ or ‘moderate’ risk of bias, the overall risk of bias was determined to be ‘moderate’ risk of bias. If at least one domain was evaluated to have either a ‘serious’ or ‘critical’ risk of bias, that study’s overall risk of bias was categorized as ‘serious’ or ‘critical’ risk of bias, respectively. Two authors (JS and HCR) independently performed the risk of bias assessment by each specific primary outcome at each of their final follow-ups, and any discrepancy was resolved through discussion and mutual consensus.

### Data synthesis and statistical analysis

For RCTs, we performed random-effects pairwise meta-analysis to calculate the weighted mean difference (WMD) between ESWT and controlled treatments (physiotherapy or rehabilitation alone) for the following patient-reported functional outcomes based on the availability of the results: Lysholm and IKDC, at 3, 6, 12, and 24 months and VAS at 6 months. If the included studies presented results with graphs without means or standard deviations, Webplotdigitizer (Webplotdigitizer v5.2) was used to extract means and standard deviations [15]. This extraction tool has been previously used for meta-analyses [16, 17] and has been reported to have higher interrater reliability than obtaining the data from the graph manually [18], as well as strong intercoder validity and reliability [19]. Moreover, when medians and interquartile ranges were only reported, the mean was presumed to be the same as the median, and IQR/1.35 was used to calculate the standard deviation [20]. Q and I^2^ statistics were calculated to evaluate the heterogeneity across the included studies for analysis [21]. The publication bias was not evaluated because the number of included studies was less than ten. All analyses were conducted using STATA Version 16 (StataCorp, LLC, College Station, TX). Other outcomes were synthesized qualitatively.

## Results

**Fig. 1:**
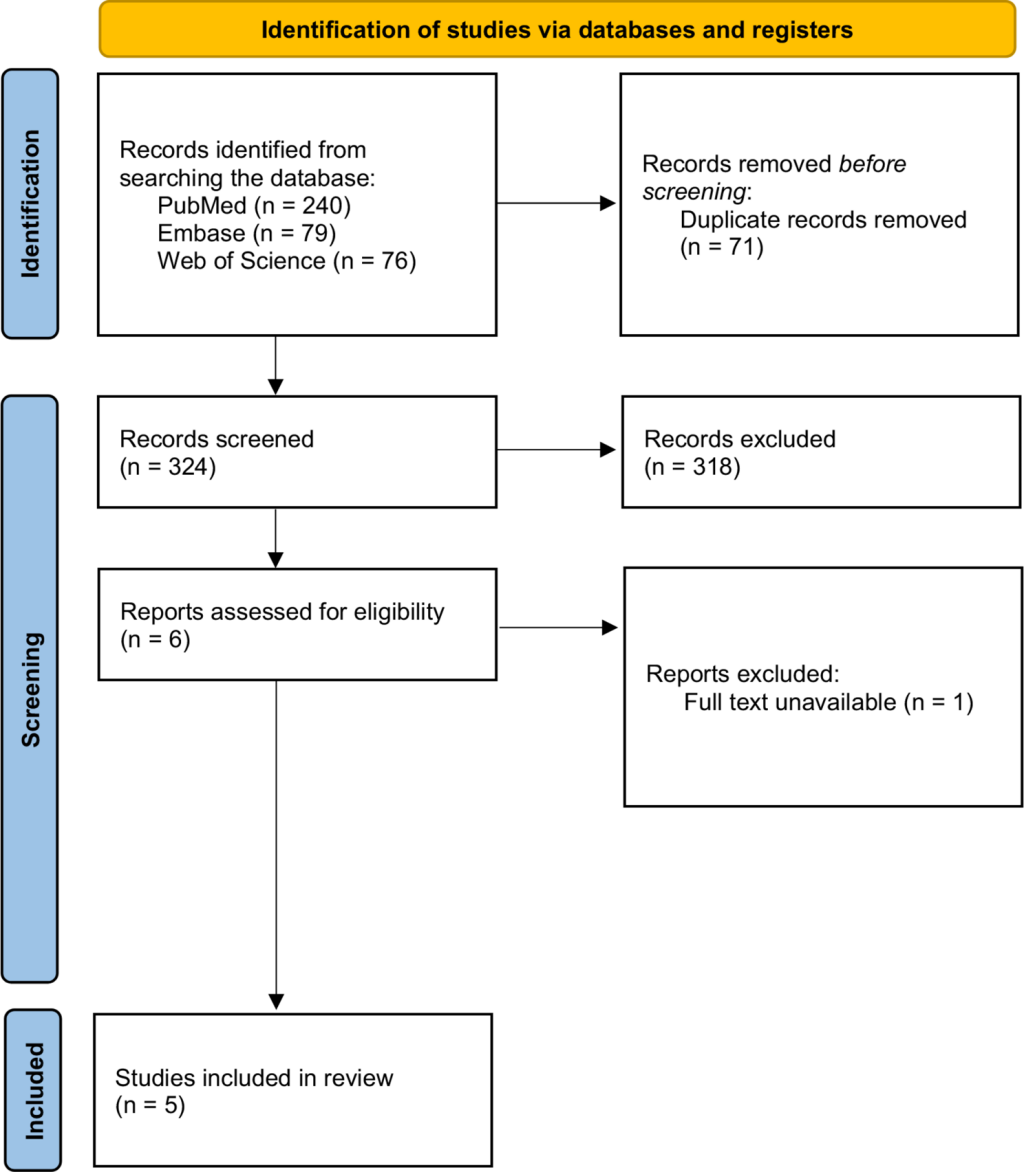
Preferred Reporting Items for Systematic Reviews and Meta-Analysis (PRISMA) flowchart showing the study selection process

The initial PubMed, Embase, and Web of Science search yielded 395 published studies–of which 71 studies were duplicates, resulting in 324 remaining studies. Then, after going through title and abstract screening, 318 studies were removed, resulting in a total of six remaining studies. These screened studies were accessed through the eligibility criteria, and one study was excluded because the full text was not available online, and its corresponding author did not respond to our inquiry regarding the full-text request. Therefore, five studies were included (Fig. 1) with 242 participants (male: 167; female: 75), with four level I studies and one level II study from Malaysia, Austria, China, and Taiwan [22–26].

While most studies did not specify the activity levels or the professional occupations of the participants, one RCT stratified the participants based on their level of play, ranging from the professional level to recreational athletes, who participated in sports at least three times per week in the middle of a season [25]. The characteristics of the included studies are summarized in Table 1. Table 3, which is included in Additional File 2, regards specific ESWT parameters and activity restrictions following ESWT, and a summary of the main findings of each study is provided in Table 4 in Additional File 3.

**Table 1:** Demographic characteristics of the included studies on implementing extracorporeal shockwave therapy (ESWT) following anterior cruciate ligament reconstruction (ACLR)

Author	Country	Study Design	Level of Evidence	Population	Symptom Duration	Graft Type	Comparator	Outcome Measurements	Follow-up Durations
Rahim 2022	Malaysia	Quasi-experimental	II	6ESWT: 9 M, mean age 28.11 [6.39] (range 21–36) 3ESWT: 9 M, mean age 23.44 [5.05] (range 20–36) Control: 7 M, mean age 29.29 [4.39] (range 22–36)	Not reported	Semitendinosus and gracilis tendon autografts (single autograft hamstring ACL reconstruction)	Physiotherapy only	Lysholm, MRI findings	6 mon
Song 2024	China	RCT	I	R-SWT: 21 M, 11 F, mean age 27.94 [6.38] Sham: 18 M, 13 F, mean age 27.00 [6.11]	Not reported	Not reported	Sham-ESWT and standard rehabilitation	Lysholm, IKDC, ROM, VAS	3-, 6-, and 24-wk
Wang 2014	Taiwan	RCT	I	F-SWT: 21 M, 5 F, mean age 28.3 [7.4] (range 15–45) Control: 21 M, 6 F, mean age 27.7 [7.7] (range 17–53)	F-SWT: 21.4 [22.5] mons (range 1–72) Control: 15.4 [21.9] mons (range 1–84)	Semitendinosus autograft	Post-operative rehabilitation only	Lysholm, IKDC, radiographic evaluation, BMD, MRI findings, and KT-1000	1 wk, 6 mon, 12 mon, and 24 mon
Weninger 2023	Austria	RCT	I	Total: 35 M, 40 F, mean age 27.65 [7.07] F-SWT: 37 patients, mean age 28.51 [7.42] Control: 28 patients, mean age 26.50 [6.52]	Not reported	Semitendinosus and gracilis tendon autografts (hamstring tendon autograft)	Rehabilitation protocol only	Return to pivoting sports, running activity, and pre-injury activity level, VAS, IKDC, Lysholm, MRI findings	3, 6, 9, and 12 mon
Zhang 2023	China	RCT	I	R-SWT: 13 M, mean age 30.5 [5.4] (range 25–42) Control: 13 M, mean age 29.2 [4.1] (range 26–39)	Not reported	Ipsilateral semitendinosus and gracilis tendon autografts (hamstring autograft)	Rehabilitation protocol only	Lysholm, IKDC, Tegner, MRI findings, knee laxity, MCID	3, 6, and 24 mon

Abbreviations: BMD, bone mineral density; ESWT, extracorporeal shockwave therapy; F, female; F-SWT, focused shockwave therapy; IKDC, International Knee Documentation Committee; M, male; MCID, minimal clinically important difference; mon, month; MRI, magnetic resonance imaging; RCT, randomized controlled trial; R-SWT, radial shockwave therapy; ROM, range of motion; VAS, visual analogue scale; wk, week

### Risk of bias assessment

**Fig. 2:**
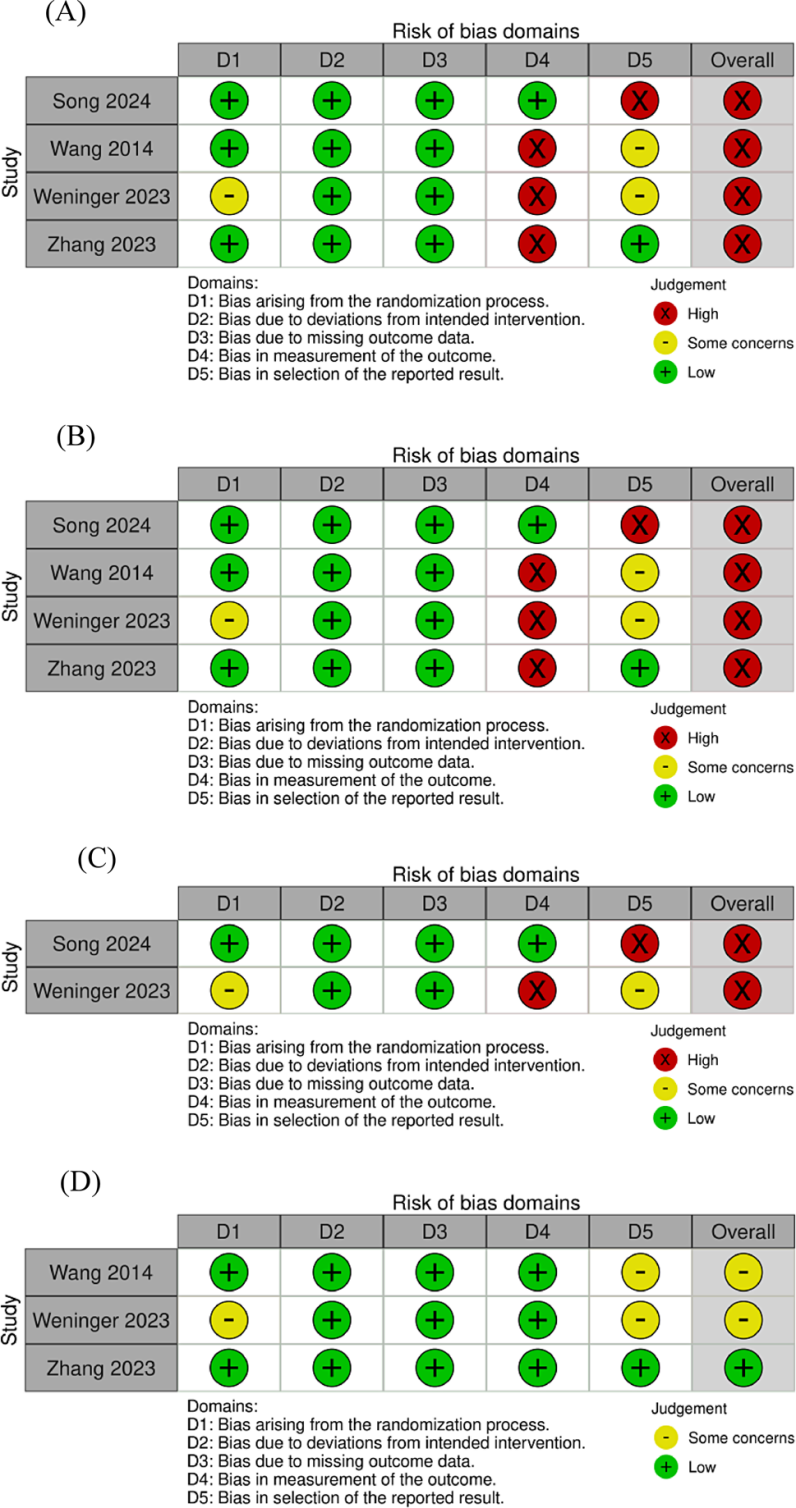
Risk of bias assessments for the (**A**) Lysholm, (**B**) International Knee Documentation Committee (IKDC), and (**C**) visual analogue scale (VAS) scores and (**D**) magnetic resonance imaging (MRI) and radiographic outcomes of randomized controlled trial (RCT) studies at the final follow-up time point

**Fig. 3:**
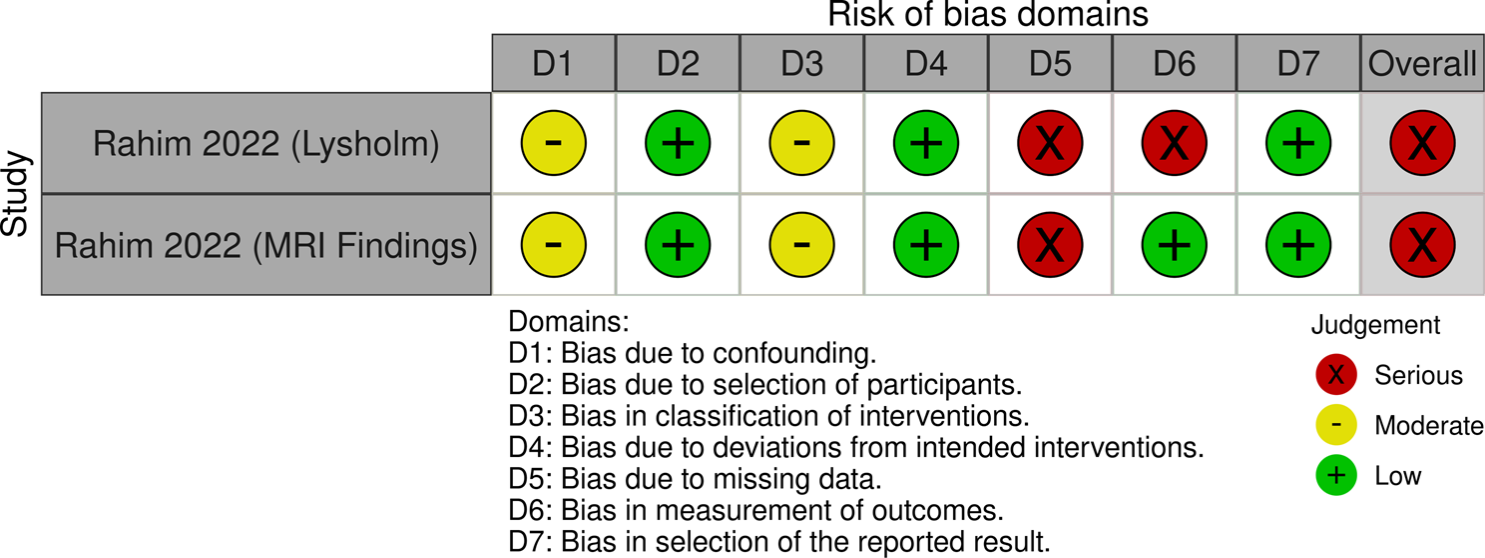
Risk of bias assessments for the Lysholm score and magnetic resonance imaging (MRI) outcomes of a non-randomized study at the final follow-up time point

Regarding the patient-reported outcomes consisting of Lysholm, IKDC, and VAS scores, all included RCTs were judged to have a ‘high’ risk of bias [23–26] (Fig. 2). For MRI and radiographic outcomes, two RCTs were considered to have ‘some concerns’ in risk of bias [23, 25] and ‘low’ for one RCT [24] (Fig. 2). One non-randomized comparative study was considered to have a ‘serious’ risk of bias for Lysholm scores and MRI outcomes [22] (Fig. 3).

### ACL graft choice

Four of the five included studies incorporated autografts. One RCT did not report the graft type used for ACLR [26]. One prospective quasi-experimental study and two RCTs utilized the semitendinosus and gracilis tendon [22, 24, 25], and one RCT implemented a single-bundle semitendinosus autograft only [23].

### ESWT characteristics

Two RCTs utilized F-SWT [23, 25] and R-SWT for two RCTs [24, 26]. One prospective quasi-experimental study used low-energy ESWT without explicitly mentioning the specific type [22].

While most studies utilized the ESWT technique post-ACLR, one RCT notably used F-SWT for a single time immediately post-ACLR while the patients were still in anesthesia (either general or spinal) from the original ACLR, with the energy flux density (EFD) of 0.298 mJ/mm^2^ [23]. Another RCT applied F-SWT starting from the fourth-week post-ACLR once a week for a total of three weeks, with the EFD of 0.25 mJ/mm^2^ [25]. Additionally, one RCT utilized R-SWT starting from the 12-week post-ACLR once a week for a total of five weeks, with the EFD ranging between 0.05 and 0.11 mJ/mm^2^ [24]. Lastly, one RCT implemented R-SWT starting from the second day post-ACLR once a week for a total of six consecutive weeks, with the EFD of 0.298 mJ/mm^2^, and the Sham-ESWT group utilized a simulated head that caused no therapeutic effect or acoustic waves with unspecified parameters [26]. Also, if the pain from R-SWT cannot be tolerated by the patient, EFD of 0.08–0.28 mJ/mm^2^ was used with a decreased number of shocks applied [26]. One prospective quasi-experimental study implemented low-energy ESWT techniques starting from the seventh-week post-ACLR once a week for a total of three or six sessions, with the EFD of 0.09 and 0.18 mJ/mm^2^, respectively, depending on the assigned intervention group [22].

One quasi-prospective study applied the low-energy ESWT at the lateral aspect of the involved knee joint [22]. One RCT applied R-SWT on the area surrounding the patella and the area approximately 10 cm above the patella to avoid the surgical site from ACLR [26], while another RCT applied it to the tibial and femoral tunnel of the knee [24]. For F-SWT, one RCT applied it to the lateral femorotibial space and tibial and lateral femoral tunnel of the knee [25], and another RCT only applied it to the tibia tunnel [23].

### Patient-reported and clinical outcomes

Among the five studies, four RCTs compared the outcome results between ACLR and the control group at different time periods [23–26]. The control groups of three RCTs underwent rehabilitation protocol only and did not receive any ESWT techniques, including sham ESWT [23–25]. However, the control group of one RCT received the sham-ESWT, a technique incorporating a simulated head to diminish interference from vibration during the therapy, resulting in no therapeutic effect and no acoustic waves [26]. One prospective quasi-experimental study compared outcomes between groups receiving either physiotherapy alone or low-energy ESWT with varying numbers of sessions (three or six) [22].

One RCT with ‘some concerns’ for risk of bias for Lysholm, IKDC, and VAS scores composed of three sessions of F-SWT once a week and a control group with only standard rehabilitation protocol reported that the improvement in IKDC, Lysholm, and VAS scores was significantly higher for the F-SWT group than for the control group (p < 0.001 for all measurements) for all follow-up periods, including 3, 6, 9, and 12 months post-ACLR [25].

In another RCT with ‘some concerns’ for risk of bias for Lysholm and IKDC scores composed of five sessions of R-SWT once a week and a control group with only standard and advanced rehabilitation protocol, Lysholm, IKDC, and Tegner scores at 24-month follow-ups showed significant improvement compared to those at 3- and 6-month follow-ups [24]. Although no between-group significance was observed for the Lysholm and Tegner scores at six months, both scores were significantly higher in the R-SWT group at 24 months [24]. However, no between-group significance was found for the IKDC score [24]. Additionally, all control and R-SWT group participants met the Minimal Clinically Important Difference (MCID) with the threshold of 8.9 at 24 months for the Lysholm score (100%, 13 of 13 patients) [24]. While all participants in the R-SWT group met MCID with the threshold of 1 point at 24 months for the Tegner score (100%, 13 of 13 patients), only 5 of 13 (38.5%) patients met MCID for the control group [24]. Moreover, 11 of 13 (84.6%) patients met MCID for IKDC with the threshold of 16.7 within the R-SWT group, while 7 of 13 (53.8%) patients met MCID for IKDC score in the control group at 24 months [24].

Additionally, one RCT with a ‘high’ risk of bias for Lysholm, IKDC, and VAS scores was composed of six sessions of R-SWT once a week, and a control group received the sham-ESWT and the standard rehabilitation [26]. This study showed that between-group significant difference was observed for Lysholm (mean ± SD; R-SWT: 66.91 ± 2.607 and sham: 61.35 ± 3.179 at 3-week; R-SWT: 75.69 ± 3.814 and sham: 69.29 ± 3.268 at 6-week), IKDC (mean ± SD; R-SWT: 42.25 ± 3.111 and sham: 37.61 ± 2.895 at 3-week; R-SWT: 49.69 ± 2.596 and sham: 41.71 ± 3.079 at 6-week), and VAS (mean ± SD; R-SWT: 1.69 ± 0.821 and sham: 2.68 ± 1.166 at 3-week; R-SWT: 0.41 ± 0.499 and sham: 1.29 ± 0.824 at 6-week) scores at three and six weeks post-ACLR [26]. However, during the 24-week follow-up period, no between-group significant difference was observed [26].

Lastly, the third RCT with ‘some concerns’ for risk of bias for Lysholm and IKDC scores consisted of F-SWT and control group with only standard post-operative rehabilitation protocol reported that a between-group significant difference was found in Lysholm scores by the F-SWT group, showing significantly improved scores compared to the control group at 12 (mean ± SD; F-SWT: 94.0 ± 4.9; Control: 87.3 ± 6.4) and 24 months (mean ± SD; F-SWT: 95.0 ± 4.6; Control: 89.0 ± 7.9) and in KT-1000 score at 24 months (mean ± SD; F-SWT: 2.4 ± 1.0; Control: 3.4 ± 1.4) [23]. However, a significant difference between groups was not found in the IKDC score [23].

For the prospective quasi-experimental study with a ‘serious’ risk of bias for Lysholm scores composed of low-energy ESWT groups undergoing three (3-ESWT) or six sessions (6-ESWT) and a control group undergoing standard physiotherapy post-operation, Lysholm scores improved significantly for all groups (6-ESWT: mean difference [MD] = 25.556, 95% confidence interval [CI] from − 38.157 to -12.954; 3-ESWT: MD = -23.556, 95% CI from − 35.471 to -11.641; control: MD = -37.143, 95% CI from − 53.828 to -20.458) at six months period, but there was no between-group significant difference observed statistically [22].

The meta-analysis of RCTs was conducted for Lysholm and IKDC scores at 3-, 6-, 12-, and 24-month follow-ups and 6-month follow-ups for VAS scores.

**Fig. 4:**
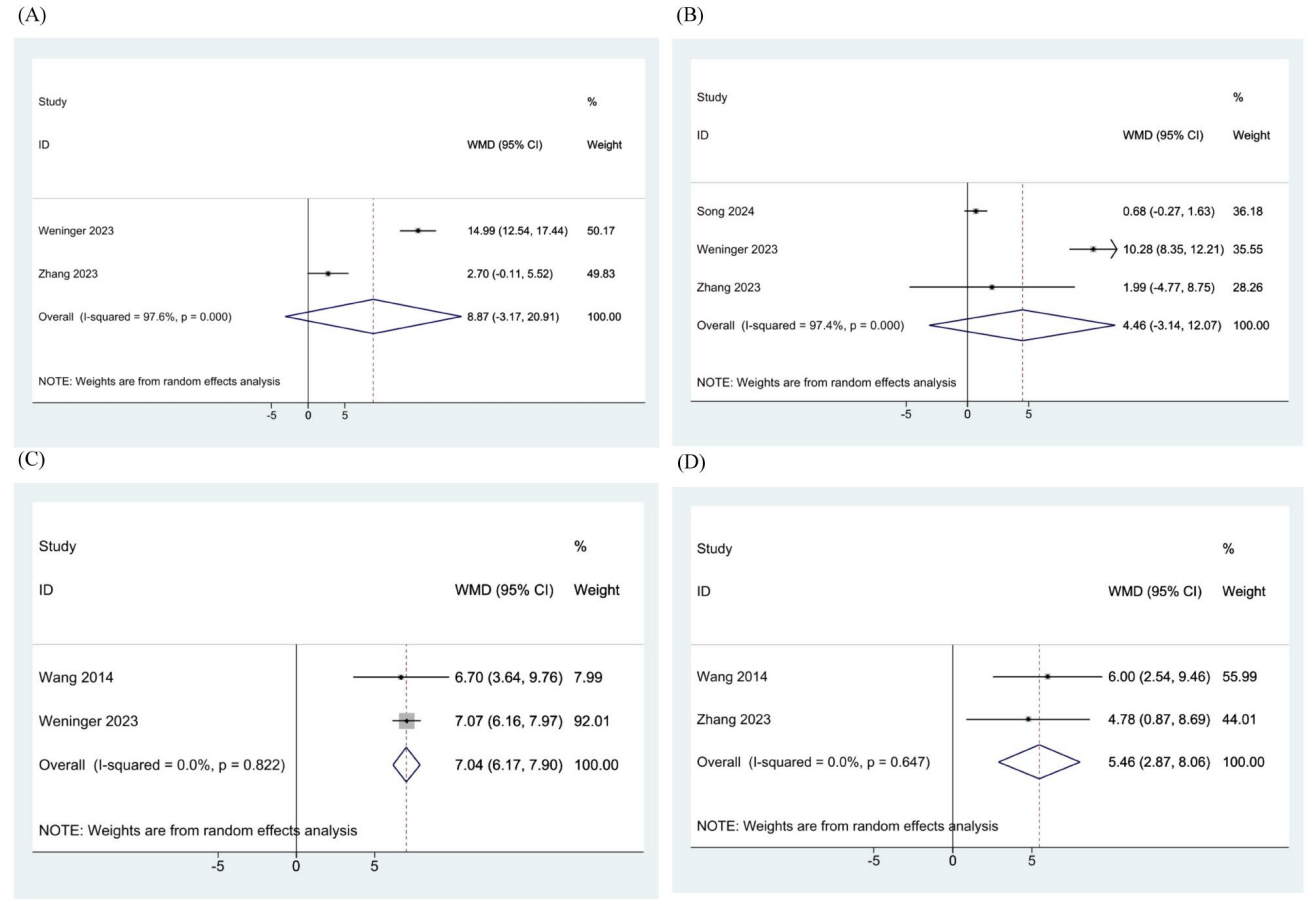
Meta-analysis of the Lysholm scores of included randomized controlled trials (RCTs) at (**A**) 3-month, (**B**) 6-month, (**C**) 12-month, and (**D**) 24-month follow-up

For Lysholm scores, the meta-analysis of three studies showed that there was no significant difference found between the ESWT group and the control group at three (WMD: 8.866, 95% CI: -3.174-20.906, I^2^: 97.6%) [24, 25] and six months (WMD: 4.463, 95% CI: -3.143-12.069, I^2^: 97.4%) [24–26]. However, the ESWT group showed a significantly better outcome at 12 (WMD: 7.037, 95% CI: 6.172–7.902, I^2^: 0%) [23, 25] and 24 months follow-up (WMD: 5.463, 95% CI: 2.870–8.056, I^2^: 0%) [23, 24] (Fig. 4).

**Fig. 5:**
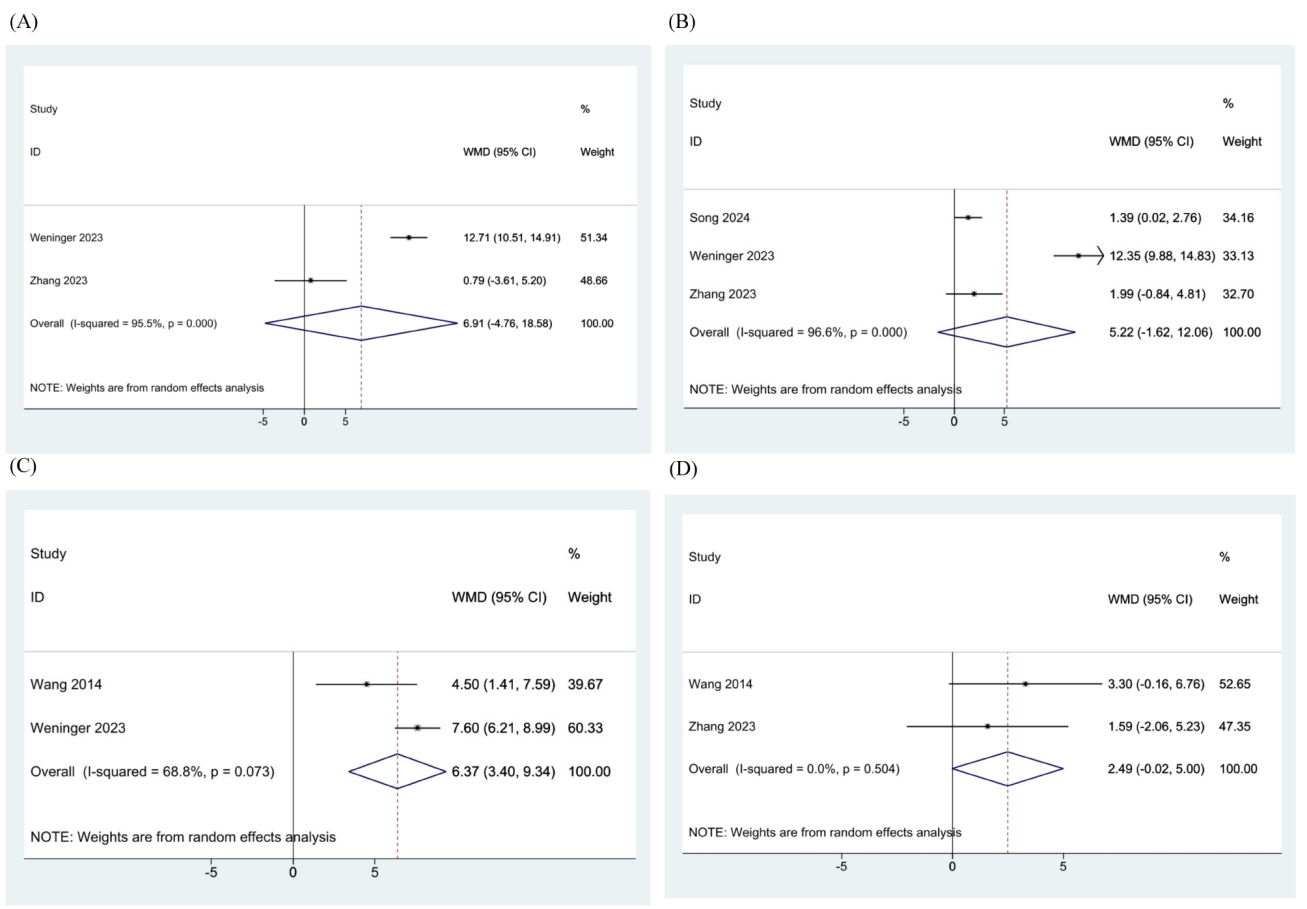
Meta-analysis of the International Knee Documentation Committee (IKDC) scores of included randomized controlled trials (RCTs) at (**A**) 3-month, (**B**) 6-month, (**C**) 12-month, and (**D**) 24-month follow-up

Regarding IKDC scores, at 12 months, the ESWT group had significantly better scores compared to the control group of each study (WMD: 6.371, 95% CI: 3.397–9.344, I^2^: 68.8%) [23, 25], while it did not at three (WMD: 6.910, 95% CI: -4.761-18.582, I^2^: 95.5%) [24, 25], six (WMD: 5.217, 95% CI: -1.624-12.058, I^2^: 96.6%) [24–26], and 24 months follow-up (WMD: 2.489, 95% CI: -0.018-4.996, I^2^: 0%) [23, 24] (Fig. 5).

**Fig. 6:**
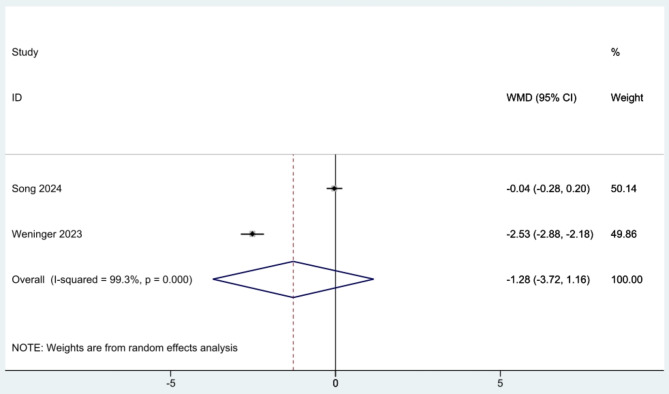
Meta-analysis of the visual analogue scale (VAS) scores of included randomized controlled trials (RCTs) at 6-month follow-up

Moreover, there was no significant difference between the two groups at six months for VAS scores (WMD: -1.280, 95% CI: -3.717-1.157, I^2^: 99.3%) [25, 26] (Fig. 6).

### MRI & radiographic findings

Four of the five included studies implemented MRI, radiographic findings, or both to determine the efficacy of ESWT following ACLR [22–25]. Song et al. did not utilize MRI or radiographic findings to identify it [26]. One prospective quasi-experimental study with a ‘serious’ risk of bias for MRI outcomes measured the incorporation of the grafts in tibial tunnels by evaluating the quality of the signals in the ligament and its connection with the bone using MRI [22]. The authors reported that the number of partially incorporated grafts in the tibial tunnel increased significantly more than that of non-incorporations for the 6-ESWT group (8 versus 1, respectively) at six months, but 3-ESWT (7 versus 2, respectively) and control groups (6 versus 1, respectively) could not meet the statistically significant improvement [22].

Zhang et al., with a ‘low’ risk of bias for MRI and radiographic outcomes, evaluated the signal strength of the ligament used for the graft and the graft’s quality by utilizing the signal-to-noise quotient (SNQ) measured from different sites [24]. Overall, the R-SWT group demonstrated lower values, indicating better outcomes compared to the control group. Regarding graft maturation, a significant improvement was reported in the tibial intraosseous graft in favor of R-SWT at six months in terms of the SNQ (*p* = 0.006), but this was not observed in the femoral intraosseous and intra-articular grafts [24]. However, during the 24-month period, no significant difference between groups was observed in the tibial intraosseous graft, whereas a significant between-group improvement was observed for the R-SWT group in both the femoral intraosseous (*p* = 0.020) and intra-articular grafts (*p* = 0.044) [24].

Similarly, Weninger et al., while noting ‘some concerns’ regarding the risk of bias for MRI and radiographic outcomes, evaluated the maturation of the ACL graft by analyzing the intensity of the signal in the posterior cruciate ligament (PCL) with the signal-intensity-ratio (SIR), which is calculated from the mean grey value in MRI, comparing that of the ACL to the PCL; the authors reported that the SIR value for F-SWT showed a significant improvement compared to the control group (F-SWT: 1.81 ± 0.88; control: 2.68 ± 1.04; p < 0.01), showing a better graft maturation for F-SWT group [25].

Wang et al., which has ‘some concerns’ regarding the risk of bias for MRI and radiographic outcomes, utilized the enlargement of the tibial tunnel from MRI that is used to evaluate the outcomes of ACLR and the bone mineral density (BMD) to determine the efficacy of the ESWT immediately post-ACLR [23]. The enlargement of the tibia tunnel of the F-SWT group decreased significantly compared to that of the control group at all time periods (6-month: p = 0.024; 24-month: p < 0.001), but there was no significant between-group difference found in BMD values [23].

### Range of motion

Song et al. determined that a between-group significant difference was observed for the range of motion (ROM) [26] at three (mean ± SD; R-SWT: 104.22 ± 8.091; sham: 94.97 ± 6.631) and six weeks (mean ± SD; R-SWT: 125.16 ± 5.941; sham: 118.48 ± 5.464) follow-up period. However, no significant difference between groups was found during the 24-week follow-up period [26]. Other RCTs and a prospective quasi-experimental study did not specify or report the ROM at different follow-up periods as their outcomes [22–25].

### Return to sports, activities, and pre-injury level

Weninger et al. reported that the mean duration to return to sports and running activity post-operative was significantly lower for the F-SWT group than the control group (return to sports: 27.92 weeks vs. 42.64 weeks, respectively, *p* < 0.001; return to running activity: 10.46 weeks vs. 18.46 weeks, respectively, *p* < 0.001), and the number of participants who returned to their pre-injury activity level post-operative was significantly higher for the F-SWT group (83.8%, 31 of 37 patients) than the control group (21.4%, 6 of 28 patients) within the follow-up duration (*p* < 0.001) [25]. Other RCTs and a prospective quasi-experimental study did not report a return to sports, activities, and pre-injury level [22–24, 26].

### Complications and adverse events

There were no reported adverse events or complications directly related to the use of ESWT for all included studies [22–26]. Regarding the complications that are not directly related to ESWT, there were quadriceps atrophy and graft failure [23], and one study excluded the patients who had minor complications from the surgery [22]. Other studies discovered that there were no complications or adverse events during the study.

## Discussion

To the best of our knowledge, our review is the first systematic review to investigate the efficacy of ESWT combined with standard rehabilitation following ACLR. All included studies showed some degree of favorable outcomes associated with the use of ESWT after ACLR in terms of patient-reported outcomes and imaging findings up to 24 months. The meta-analyses of included RCTs also demonstrated that ESWT combined with rehabilitation may result in better Lysholm and IKDC scores at 12 months and Lyholm scores at 24 months compared to standard rehabilitation alone.

Several patient-reported outcome measures have been developed and used to capture functional outcomes after ACLR [27]. In the included studies, Lysholm, IKDC, and Tegner were reported as favorable to ESWT groups compared to their control groups [22–26]. These outcomes indicate the amount of recovery following ACLR by evaluating the functionality, stability, symptoms, activity level of sports, and symptoms of the knee [28–30]. At a follow-up period of less than 12 months, there was some variability in results regarding the patient-reported outcomes. In the short term, Song et al. noted that the ESWT group had significantly better outcomes in Lysholm, IKDC, and VAS at three- and six-week post-operative than the control group [26]. On the other hand, during the follow-up period of six months, Song et al. and Rahim et al. found no statistically significant difference between ESWT and control groups in Lysholm, IKDC, and VAS for Song et al. and Lysholm for Rahim et al. [22, 26]. Zhang et al. also reported no statistically significant difference in IKDC, Tegner, and Lysholm scores at the three- and six-month post-operative periods between the ESWT group and the control group [24]. The meta-analysis results of Lysholm, IKDC, and VAS scores demonstrated similar findings in that there was no significant difference between the ESWT control groups at 3- and 6-month follow-up for Lysholm and IKDC scores and 6-month follow-up for VAS scores.

Interestingly, however, in the longer term, equal to or beyond 12 months, favorable patient-reported outcomes were seen with the use of ESWT. Weninger et al. reported that the ESWT group had significantly better IKDC, Lysholm, and VAS compared to the control group at 12 months [25]. Moreover, Wang et al. reported that Lysholm score was significantly better than the control group for the ESWT group at both 12 and 24 months, although no significant difference was seen for IKDC at those follow-up periods [23]. Furthermore, while most studies did not compare the results of each patient-reported outcome to their MCIDs, Zhang et al. reported that the ESWT group not only met the MCID for Lysholm, Tegner, and IKDC scores but also had more patients meeting the MCID for Tegner and IKDC scores compared to the rehabilitation-only group at 24 months, although the statistical significance testing regarding the number of patients exceeding MCID between groups was not conducted [24]. Similarly, at 12- and 24-month follow-up for the Lysholm score and 24-month follow-up for the IKDC score, the results of our meta-analysis indicated that using ESWT in addition to standard rehabilitation led to significantly better outcomes than the control group. However, according to the MCID thresholds for Lysholm (8.9) and IKDC scores (16.7) [24, 31], the WMDs of these outcomes did not exceed the MCID thresholds, meaning that the clinical significance of the usage of ESWT following ACLR is still questionable. Additionally, there was no significant difference between the two groups for IKDC scores at the 24-month follow-up from our meta-analysis [23, 24]. Despite these results, given that ESWT is a non-invasive and safe intervention that has shown promising results in other orthopaedic conditions [5, 32], it can be considered with standard rehabilitation to improve patient-reported outcomes.

In one study, Wang et al. implemented a KT-1000 arthrometer to objectively evaluate knee laxity [23], using an absolute value measured from the identical knee before and after ACLR. The KT-1000 arthrometer allows the rater to measure the amount of the anterior movement of the tibia respective to the femur [33], and its side-to-side difference greater than 5 mm is considered a loose knee or a surgical failure [34, 35]. During the final follow-up at two years post-operative period, the ESWT group had a significantly better KT-1000 score compared to the control group, although there was no significant difference observed in other follow-up periods [23]. However, previous studies mentioned that KT-1000 is instead a dichotomous diagnostic tool for ACL injuries, not a continuous outcome [33, 36]. Moreover, many studies often use the side-to-side difference of knee laxity measured by KT-1000 on both injured and uninjured knees as an outcome to evaluate surgical outcomes [34, 35, 37, 38], while Wang et al. did not [23]. Also, when its side-to-side difference measured by the KT-1000 arthrometer exceeds 5 mm, it was not clinically significant in the long term since this difference was not associated with extra procedures or worse clinical outcomes [34]. Therefore, this result should be interpreted with caution.

Four studies utilized MRI and radiographic findings to evaluate the clinical improvement following ESWT treatment after ACLR, including graft incorporation and maturation [22–25]. Various measurement techniques were used to analyze graft incorporation and maturation, such as SIR [25] and signal intensity and quality from the graft [22, 24]. Rahim et al. reported that the ESWT group, which had six sessions of ESWT, had significantly more patients with grafts incorporated compared to non-incorporated grafts at six months, but there was no significant difference with the group that underwent three ESWT sessions or physiotherapy alone [22]. However, no statistical testing was conducted to evaluate whether there was a significant difference in the number of incorporated grafts between the control and the ESWT groups at six months [22]. Moreover, Weninger et al. reported that graft maturation appeared significantly better for the ESWT group than the control group at 12 months [25]. For Zhang et al., although there was no statistically significant difference at 24 months regarding the graft maturation measured at the tibial side, the graft maturation was significantly better when measured at the femoral and intra-articular side at 24 months [24]. Similarly, Wang et al. used the ratio of the area of the graft and the screw for connecting the ends of the graft to calculate the autograft integration as a surrogate measure of graft maturation [23]. At six and 24 months, autograft integration was significantly better for the ESWT group than the control group [23]. The graft maturation has been suggested to be a sign of graft ligamentization [25], which is the process of mechanical remodeling to adapt to the new environment to develop as the original ACL [39, 40]. Previous studies have mentioned that both graft incorporation and ligamentization processes continue until the utilized graft during the ACLR achieves properties like the original ACL, reflecting successful post-operative rehabilitation [39]. Based on the limited studies, ESWT combined with standardized rehabilitation was associated with better graft maturation and incorporation beyond 12 months. However, more studies are needed to confirm these findings and whether these imaging findings correlate with patient-reported outcomes.

Return to sport following ACLR may take up from nine months to 12 months [41, 42], and the reinjury rate of ACL after the reconstruction ranges from 1.5 to 37.5% [43]. Given the variable range of reinjury rates, there has been much interest in research attempting to optimize recovery through rehabilitation and adjunctive therapies following ACLR. One study examining strategies for enhancing the osteointegration of grafts adopted from tendons discovered that biological techniques, including gene transfers and stem cell transplantation, bone substitution technique, and physical stimulation methods such as shockwave and low-intensity pulsed ultrasound (LIPUS) treatments, yield positive results in osteointegration, an essential indicator for long-term clinical outcomes following ACLR [44]. Previous studies have reported that shockwave therapy may lead to neovascularization at the junction of the bone and tendon and increase the mass and strength of the bone dose-dependently, which improves the osteointegration of grafts following ACLR [44–46]. Furthermore, another study reviewing various approaches to graft healing post-ACLR suggested that implementing biological interventions such as ESWT, platelet-rich plasma, and calcium-phosphate-hybridized tendon could lead to positive outcomes with reported mixed outcomes regarding remnant repairs and bone substitutes [47]. The results of our systematic review support the implementation of ESWT as a potential adjunctive therapy to standard rehabilitation post-ACLR to optimize recovery.

The mechanism of how ESWT may improve graft incorporation is not completely understood. However, given that ESWT promotes neovascularization [22–24], better blood flow at the junction of tendon-bone [22], the function of the knee joint [26], and osteogenesis [24], these mechanisms may partially explain the favorable results related to graft incorporation and maturation using ESWT post-ACLR. ESWT may also contribute to better graft incorporation by inducing interstitial and extracellular responses such as pain relief [6], enhanced collagen generation, and increased cell growth and tissue repair [7]. It has also been reported that the rudimentary aspects of ACLR involve cells’ growth, movement, and differentiation from the remaining ACL tissue and nearby areas, and ESWT may be an effective approach to enhance those processes and promote biological activities of ACL remnant for better graft maturation [48].

ESWT has also been incorporated in recovery after other orthopaedic surgeries other than ACLR. Up to date, ESWT has been used in knee arthroscopic surgery, calcifying tendonitis arthroscopy, rotator cuff repair, and total knee arthroplasty [49–52], but two studies reported that it was ineffective for calcifying tendonitis arthroscopy [50] and rotator cuff repair [51]. Moreover, a systematic review identified that the usage of ESWT in knee arthroscopic surgery would be premature, given the limited evidence available [49]. Previous studies regarding the use of ESWT on the knee reported that no major adverse events occurred during or after the use of ESWT on the knee, and studies in this review reported only minor adverse events [5, 22–26, 53, 54]. Any potential adverse effects of ESWT include pain in the applied area, transitory erythema, skin bruising, and nausea, which are still minor [5, 32]. While further investigation is needed to confirm the efficacy of ESWT following other orthopaedic surgeries, it is a safe procedure without major complications and may have a potential role in improving post-surgical outcomes, including ACLR.

This review has several limitations. First, only five studies were included in this review, and most of these studies had some concerns or a high risk of bias. Specifically, regarding the primary patient-reported outcomes, four RCTs were determined to have a ‘high’ risk of bias [23–26] and a ‘serious’ risk of bias in one prospective quasi-experimental study [22]. Furthermore, four studies were published in Asia [22–24, 26] and one in Europe [25]. Thus, the results of our review may not be generalized to other patient populations. Second, given different follow-up periods for different outcomes across the studies, only two or three RCTs were included for each meta-analysis. Third, the number of ESWT sessions, types of ESWT devices, and ESWT parameters differed across the studies, and therefore, it is difficult to make recommendations for the ideal ESWT type and setting for patients undergoing ACLR. Additionally, only one study implemented sham shockwave to blind its patients [26]. Future trials may consider incorporating sham shockwave to ensure patient blinding and comparing different devices or parameters to optimize the ESWT protocol.

Given these limitations, the results of our review should be cautiously interpreted. High-quality RCTs, with proper trial protocol registration and management of outcome measurements and missing data, would be necessary to substantiate the findings of our study further. Future studies can also be improved with the use of objective outcome measures, such as return to sports or sports performance metrics, rather than relying on subjective patient-reported outcomes. This process will allow for a more thorough analysis and understanding of the effectiveness of the ESWT in addition to the standard rehabilitation after ACLR.

## Conclusion

ESWT combined with standard rehabilitation may potentially lead to better patient-reported outcomes, including Lysholm and IKDC scores and graft maturation. However, a cautious interpretation of this study is needed as only five studies were included, and long-term outcomes were unavailable for a few studies. Further high-quality studies are needed to confirm the findings of our review.

## Electronic supplementary material

Below is the link to the electronic supplementary material.

**Additional file 1: Table 2.** Search strategies. Search terms used from each database were listed

**Additional file 2: Table 3.** ESWT and co-intervention characteristics of the included studies. ESWT parameters, co-interventions, adverse events, and activity restrictions following ESWT were listed for each included study

**Additional file 3: Table 4.** Summary of main findings of the included studies. The main results and return to sports of each study have been explained

## Data Availability

Any data utilized in this study are available upon reasonable request from the corresponding author.

## Abbreviations

ACL: Anterior cruciate ligament
ACLR: Anterior cruciate ligament reconstruction
BMD: Bone mineral density
CI: Confidence interval
EFD: Energy flux density
ESWT: Extracorporeal shockwave therapy
F-SWT: Focused shockwave therapy
IKDC: International Knee Documentation Committee
MCID: Minimal clinically important difference
MD: Mean difference
MRI: Magnetic resonance imaging
PRISMA: Preferred Reporting Items for Systematic Reviews and Meta-Analyses
RCT: Randomized controlled trial
R-SWT: Radial shockwave therapy
RoB 2: a revised Cochrane risk-of-bias tool
ROBINS-I: Risk of Bias in Non-randomised Studies of Interventions
ROM: Range of motion
SIR: Signal intensity ratio
VAS: Visual analogue scale
WMD: Weighted mean difference
