# Supplementary material for: Use of extracorporeal shockwave therapy combined with standard rehabilitation following anterior cruciate ligament reconstruction: a systematic review with meta-analysis
Source: BMC Musculoskelet Disord. 2025 Jan 23;26:79. doi: 10.1186/s12891-025-08277-9 (PMC11756125; doi:10.1186/s12891-025-08277-9)
Supplement: Supplementary file 1 — Additional file 1: Table 2. Search strategies. Search terms used from each database were listed [file 12891_2025_8277_MOESM1_ESM.docx]

| Table 2. Search Strategies | |  |
| --- | --- | --- |
| **Database** | **Search terms** | **Number of results** |
| **PUBMED (NLM)** | (("Extracorporeal Shockwave Therapy"[Mesh]) OR (Shockwave*[Title/Abstract] OR "shock wave*"[Title/Abstract] OR ESWT[Title/Abstract] OR “focused shockwave”[Title/Abstract] OR “radial shockwave”[Title/Abstract])) AND ((ACL[Title/Abstract] OR "anterior cruciate ligament"[Title/Abstract] OR "anterior cruciate ligament reconstruction"[Title/Abstract] OR "ACL reconstruction"[tiab:~3] OR "ACL injury"[tiab:~3] OR “Knee Injury”[tiab:~3] OR “Knee Ligament Reconstruction”[tiab:~3] OR “Knee Surgery”[tiab:~3] OR Knee*[tiab]) OR ("Anterior Cruciate Ligament"[Mesh] OR "Anterior Cruciate Ligament Reconstruction"[Mesh])) | 240 |
| **EMBASE (ELSEVIER)** | #1 ​​('Shockwave therapy'/exp) OR (shockwave* OR 'shock wave*' OR ESWT OR ‘extracorporeal shockwave’ OR ‘radial shockwave’ OR ‘focused shockwave’):ti,ab,kw  #2 ('anterior cruciate ligament'/exp OR 'ACL' OR 'anterior cruciate ligament reconstruction'/exp OR 'anterior cruciate ligament injury'/exp OR 'anterior cruciate ligament rehabilitation' OR 'knee injury'/exp OR 'knee ligament reconstruction' OR 'knee surgery'/exp) #1 and #2 | 76 |
| **WEB OF SCIENCE (CLARIVATE)** | ALL=(("Extracorporeal Shockwave Therapy") OR (Shockwave* OR "shock wave*” OR ESWT OR “focused shockwave” OR “radial shockwave”)) AND ALL=((ACL OR ("anterior cruciate ligament") OR ("anterior cruciate ligament reconstruction") OR ("ACL reconstruction") OR ("ACL injury") OR (“Knee Injury”) OR (“Knee Ligament Reconstruction”) OR (“Knee Surgery”) OR ("Anterior Cruciate Ligament") OR ("Anterior Cruciate Ligament Reconstruction”))) | 79 |
