# Supplementary material for: Use of extracorporeal shockwave therapy combined with standard rehabilitation following anterior cruciate ligament reconstruction: a systematic review with meta-analysis
Source: BMC Musculoskelet Disord. 2025 Jan 23;26:79. doi: 10.1186/s12891-025-08277-9 (PMC11756125; doi:10.1186/s12891-025-08277-9)
Supplement: Supplementary file 2 — Additional file 2: Table 3. ESWT and co-intervention characteristics of the included studies. ESWT parameters, co-interventions, adverse events, and activity restrictions following ESWT were listed for each included study [file 12891_2025_8277_MOESM2_ESM.docx]

| Table 3. ESWT and co-intervention characteristics of the included studies | | | | | | |
| --- | --- | --- | --- | --- | --- | --- |
| **Author** | **Country** | **Study Design** | **ESWT Parameters** | **Activity Restriction Following ESWT** | **Co-intervention** | **Adverse Events** |
| Rahim 2022 | Malaysia | Quasi-experimental | ShockMaster 300 (GymnaUniphy, Germany) 6-ESWT: EFD of 0.18 mJ/mm2; 500 shocks; once per wk; total 6 wks 3-ESWT: EFD of 0.09 mJ/mm2; 500 shocks; once per wk; total 3 wks; no anesthesia used | Not reported | Normal physiotherapy rehabilitation, knee braces and crutches | No adverse events reported related to ESWT |
| Song 2024 | China | RCT | EMS Swiss Dolor-Clast R-SWT: EFD of 0.298 mJ/mm2 at 6-8 Hz (0.08-0.28 mJ/mm2 if pain cannot be tolerated); 2500 shocks total (1200-1500 shocks total if pain cannot be tolerated); once a wk; total 6 wks; no anesthesia reported Sham: simulated head causing no therapeutic effect or acoustic waves; no parameter explicitly stated | Not reported | Preoperative rehabilitation and postoperative standard rehabilitation | No adverse events reported related to ESWT |
| Wang 2014 | Taiwan | RCT | OssaTron (SANUWAVE, USA) EFD of 0.298 mJ/mm2; 1500 shocks; immediately post-ACLR; under general or spinal anesthesia | After 6 wks, patients were permitted to bear their full weight. | Post-operative rehabilitation for three to six wks. | No adverse events reported related to ESWT.  Other complications exist that are not directly related to ESWT, such as quadriceps atrophy exceeding 2.0 cm, donor site morbidity, and graft failure. |
| Weninger 2023 | Austria | RCT | Duolith SD1 Ultra (Storz Medical AG, Switzerland) EFD of 0.25 mJ/mm2 at 5 Hz; 1500 shocks total; once per wk; total 3 wks; no anesthesia reported | Not reported | Rehabilitation protocol after surgery adapted from "Early Active Rehabilitation" | No adverse events reported related to ESWT |
| Zhang 2023 | China | RCT | EMS Swiss Dolor-Clast EFD of 0.05-0.11 mJ/mm2 at 6-8 Hz; 2000 shocks total; once a wk; total 5 wks; no anesthesia reported | Not reported | Standard (0-12 wks post-ACLR) and advanced rehabilitation protocol (3 mons post-ACLR) | No adverse events reported related to ESWT |
| Abbreviations: ACLR, anterior cruciate ligament reconstruction; EFD, energy flux density; ESWT, extracorporeal shockwave therapy; mon, month; RCT, randomized controlled trial; R-SWT, radial shockwave therapy; SIR, signal intensity ratio; wk, week. | | | | | | |
