# Supplementary material for: Use of extracorporeal shockwave therapy combined with standard rehabilitation following anterior cruciate ligament reconstruction: a systematic review with meta-analysis
Source: BMC Musculoskelet Disord. 2025 Jan 23;26:79. doi: 10.1186/s12891-025-08277-9 (PMC11756125; doi:10.1186/s12891-025-08277-9)
Supplement: Supplementary file 3 — Additional file 3: Table 4. Summary of main findings of the included studies. The main results and return to sports of each study have been explained [file 12891_2025_8277_MOESM3_ESM.docx]

| Table 4. Summary of main findings of the included studies | | | | |
| --- | --- | --- | --- | --- |
| **Author** | **Country** | **Study Design** | **Main Findings** | **Return to Sports/Activities** |
| Rahim 2022 | Malaysia | Quasi-experimental | A number of grafts with partial incorporation was significantly higher than non-incorporation for 6ESWT group at 6 mon, but other groups did not. Lysholm scores improved significantly for all groups at 6 mon, but there was no between-group significant difference observed. Lysholm scores improved significantly for all groups (6-ESWT: MD = 25.556, 95% CI from -38.157 to -12.954; 3-ESWT: MD = -23.556, 95% CI from -35.471 to -11.641; control: MD = -37.143, 95% CI from -53.828 to -20.458) at six months period, but there was no between-group significant difference observed statistically. | Not reported |
| Song 2024 | China | RCT | Between-group significant difference was observed for Lysholm, ROM, IKDC, and VAS scores at three and six weeks post-ACLR. However, at 24-week follow-up period, no between-group significant difference was observed. | Not reported |
| Wang 2014 | Taiwan | RCT | Between-group significant difference was found in Lysholm score by F-SWT group showing better score than the control group at 12 and 24 mons. It was also seen by F-SWT group having a better value than the control group in KT-1000 at 24 mons. For radiographic evaluation on tibial tunnel, F-SWT group had a significantly smaller value than that of the control group. For MRI findings on tibia tunnel enlargement, F-SWT group decreased significantly than that of the control group. No between-group significant difference found in IKDC score and BMD values. | Not reported |
| Weninger 2023 | Austria | RCT | Return to pivoting sports and running activity was significantly lower for F-SWT group than the control group.  The number of participants who returned to their pre-injury activity level was signficantly higher for F-SWT group than the control group within the follow-up duration.  Significant improvement in IKDC, Lysholm score, and VAS for F-SWT group was seen than those of the control group at 3-, 6-, 9-, and 12-mon follow-up period.  Mean SIR for F-SWT group was significantly lower than that of the control group. | Return to pivoting sports:  F-SWT: 27.99 [2.99] wks Control: 42.64 [5.18] wks Return to running activity: F-SWT: 10.46 [1.48] wks Control: 18.46 [3.28] wks  Return to pre-injury activity level: F-SWT: 31/37 participants Control: 6/28 participants |
| Zhang 2023 | China | RCT | Lysholm, IKDC, and Tegner scores at 24-mon follow-up was significantly improved compared to that of at 3- and 6-mon follow-up.  No between-group significance for Lysholm and Tegner scores found at 6-mon, but it was observed at 24-mon for both scores, with R-SWT having significantly higher score. No between-group significance found for IKDC score. All participants in both control and R-SWT group met MCID at 24-mon for Lysholm score. All participants in R-SWT group met MCID at 24 mon for Tegner score, but 5/13 met it for control group. For IKDC, 11/13 met MCID in R-SWT group, while 7/13 met MCID in R-SWT group. No significant difference found for the anterior tibial translation's side-to-side difference between and within groups at follow-up period. For graft maturation, a significant difference was found in the tibial intraosseous graft but not in the femoral intraosseous and intra-articular graft at 6-mon in terms of the signal-to-noise quotients. During 24-mon, no significant difference was found on the tibial intraosseous graft, but a significant difference was found in the femoral intraosseous and intra-articular graft at 6-mon in terms of the signal-to-noise quotients. The R-SWT group had lower values. | Not reported |
| Abbreviations: ACLR, anterior cruciate ligament reconstruction; BMD, bone mineral density; ESWT, extracorporeal shockwave therapy; F-SWT, focused shockwave therapy; IKDC, International Knee Documentation Committee; MCID, minimal clinically important difference; mon, month; MRI, magnetic resonance imaging; RCT, randomized controlled trial; R-SWT, radial shockwave therapy; ROM, range of motion; SIR, signal intensity ratio; VAS, visual analogue scale; wk, week. | | | | |
